# Celiac Vagus Nerve Stimulation Recapitulates Angiotensin II-Induced Splenic Noradrenergic Activation, Driving Egress of CD8 Effector Cells

**DOI:** 10.1016/j.celrep.2020.108494

**Published:** 2020-12-15

**Authors:** Lorenzo Carnevale, Fabio Pallante, Marialuisa Perrotta, Daniele Iodice, Sara Perrotta, Stefania Fardella, Francesco Mastroiacovo, Daniela Carnevale, Giuseppe Lembo

**Affiliations:** 1 Department of AngioCardioNeurology and Translational Medicine, I.R.C.C.S. INM Neuromed, 86077 Pozzilli (IS), Italy; 2 Department of Molecular Medicine, “Sapienza” University of Rome, 00161 Rome, Italy

**Keywords:** angiotensin II, bioelectronic medicine, neuroimmune interface, vagus nerve stimulation, neural circuits

## Abstract

Angiotensin II (AngII) is a peptide hormone that affects the cardiovascular system, not only through typical effects on the vasculature, kidneys, and heart, but also through less understood roles mediated by the brain and the immune system. Here, we address the hard-wired neural connections within the autonomic nervous system that modulate splenic immunity. Chronic AngII infusion triggers burst firing of the vagus nerve celiac efferent, an effect correlated with noradrenergic activation in the spleen and T cell egress. Bioelectronic stimulation of the celiac vagus nerve, in the absence of other challenges and independently from afferent signals to the brain, evokes the noradrenergic splenic pathway to promote release of a growth factor mediating neuroimmune crosstalk, placental growth factor (PlGF), and egress of CD8 effector T cells. Our findings also indicate that the neuroimmune interface mediated by PlGF and necessary for transducing the neural signal into an effective immune response is dependent on α-adrenergic receptor signaling.

## Highlights

• Bioelectronic stimulation of celiac vagus nerve primes a splenic immune response
• Vagus nerve stimulation selectively drives the egress of CD8+ effector T cells
• Placental growth factor (PlGF) is a key mediator of the splenic neuroimmune coupling
• Vagus nerve stimulation induces splenic PlGF through α-adrenergic receptors signaling

## Graphical Abstract

**Figure:**
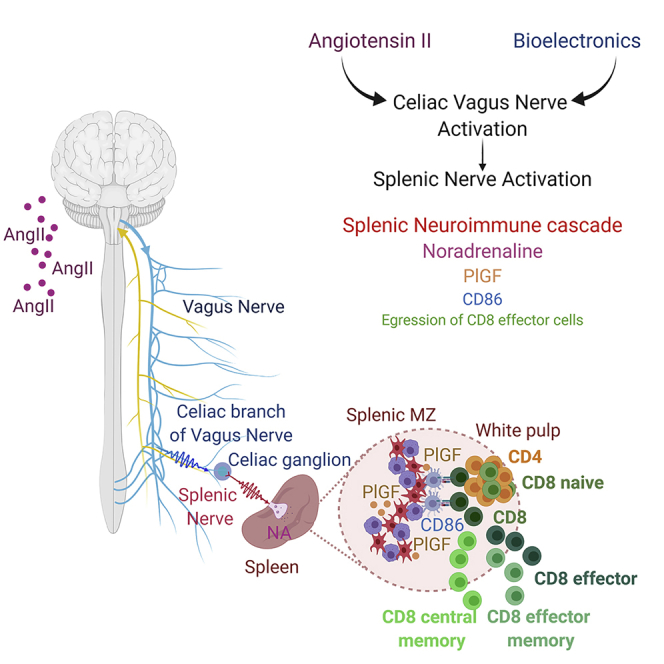

## Teaser

Carnevale et al. show that stimulation of the celiac vagus nerve primes an immune response in the spleen, driving selective egress of CD8^+^ T cells. This is mediated by placental growth factor, whose expression in the spleen is regulated by a α-adrenergic receptor signaling.

## Introduction

Angiotensin II (AngII) is a peptide hormone with potent cardiovascular effects that are either induced through the cognate receptors expressed in the cardiovascular system or mediated by central actions that in turn recruit the autonomic nervous system (ANS) (Forrester et al., 2018). More recently, it has become clear that AngII exerts additional immune-modulating functions capable of influencing the cardiovascular system (Drummond et al., 2019). Actually, the lack of lymphocytes, and more specifically of CD8 T cells, hinders the classical hypertensive response evoked by chronic AngII exposure in mice (Guzik et al., 2007; Trott et al., 2014). Although the immune-modulating functions of AngII are still being investigated, the splenic reservoir is known to respond to the peptide hormone. In fact, we and others have described both direct effects of AngII on the splenic immune reservoir (Cortez-Retamozo et al., 2013; Epelman et al., 2014; Swirski et al., 2009) and indirect effects mediated by central actions (Carnevale et al., 2014, 2016; Marvar et al., 2010).

The spleen is a secondary lymphoid organ densely innervated by noradrenergic fibers, departing from the left celiac ganglion through the splenic nerve, and hence subjected to sympathetic influences (Carnevale et al., 2014; Felten and Olschowka, 1987). The ANS helps physiologically regulate the cardiovascular system. The classical view of the ANS identifies its two main arms, the sympathetic and parasympathetic nervous systems, as key modulators of blood pressure, heart rate, vascular tone, and renal function (Abboud, 1982). Yet one efferent arm of the ANS, directed to the spleen, has also been considered a critical modulator of immune responses (Bassi et al., 2020; Chavan et al., 2017; Ordovas-Montanes et al., 2015).

Building on this, our group demonstrated that the ANS plays a pivotal role in modulating the immune response involved in hypertension (Carnevale et al., 2014, 2016). Significantly, we observed that the left celiac ganglion is a crucial crossroads of the hypertensive response to AngII. We know that this hormone activates a splenic sympathetic drive that, by promoting the release of placental growth factor (PlGF), an angiogenic growth factor in the splenic parenchyma, primes the adaptive immune system (Carnevale et al., 2014, 2016). Furthermore, we showed that splenic nerve activation by AngII is disrupted in the absence of an intact left celiac vagus nerve (Carnevale et al., 2016), thus suggesting the existence of a direct connection between these two arms of the ANS. In addition, AngII recruitment of this pathway effectively activated T cell priming and egress toward high blood pressure target organs, such as the vasculature and kidneys (Carnevale et al., 2016). Disrupting specific brain areas in the circumventricular organs, rendering them incapable of sensing circulating AngII, also produced the same effect (Marvar et al., 2010). How do these different parts of the ANS interact when AngII levels are chronically increased? In this study, we aimed to investigate the neuroimmune interface by analyzing the vagal influences on splenic sympathetic nervous activity (SSNA) during chronic exposure to AngII and, more important, by determining if a bioelectronic intervention in the vagus-mediated parasympathetic drive can handle the splenic immune response.

## Results

### AngII Activates Celiac Vagus Nerve Activity

**Figure 1:**
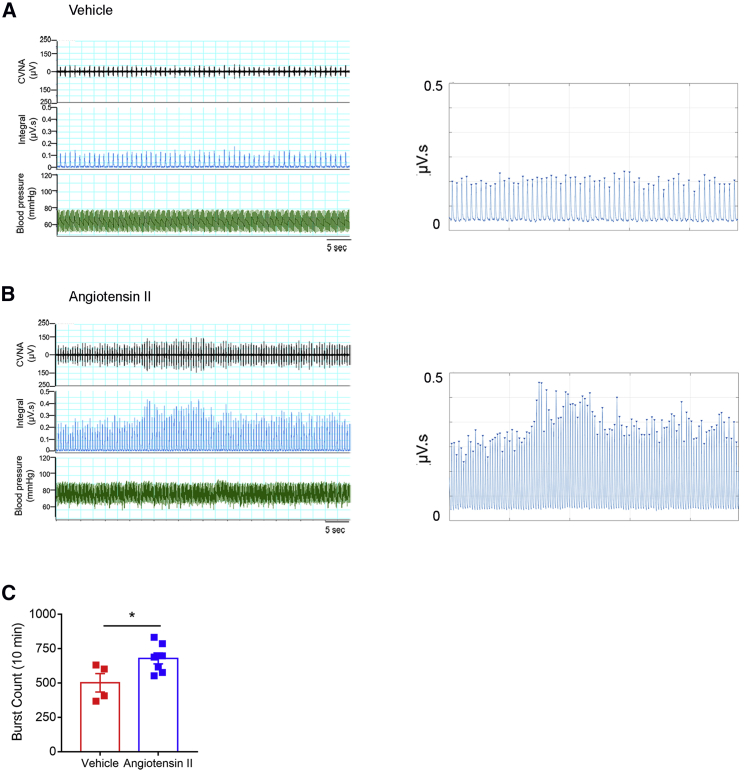
Angiotensin II Increases Celiac Vagus Nerve Activity (A and B) Representative raw, integrated signal, and blood pressure signal (left panels), with a highlight of the burst count procedure on the integrated signal carried out by MATLAB (right panels). (C) Burst count, measured from integrated signal, was significantly increased in mice subjected to AngII infusion compared with vehicle (Veh) (t(9) = 2.451, ^∗^p < 0.05). Data were obtained from n = 4 Veh and n = 7 AngII mice and are represented as mean ± SEM.

To elucidate the vagus nerve efferent celiac branch’s direct involvement in AngII-induced activation of the splenic nerve, we directly recorded and then analyzed the celiac vagus nerve activity (CVNA). Mice were implanted with osmotic minipumps containing AngII, or vehicle as control, for 3 days. We have previously shown that in this time window mice are in a pre-hypertensive phase, with blood pressure levels comparable with those of vehicle-treated mice (Carnevale et al., 2014, 2016). However, 3 days of AngII are enough to enhance the sympathetic nervous system (SNS) in the spleen, as evidenced by increased tyrosine hydroxylase (TH), the rate-limiting enzyme for noradrenaline synthesis, in the marginal zone marked by CD169-positive macrophages (Figure S1A, related to Figure 1). This SNS overdrive was coupled with a significantly reduced area of CD3-positive cells in the spleen, suggesting immune activation and egress (Figure S1B, related to Figure 1).

Next, we used a bipolar electrode to record CVNA at the same time point after AngII or vehicle infusion (Figure 1). Representative recordings of CVNA clearly demonstrate more burst activity above the background noise in AngII-infused mice compared with vehicle-treated mice (Figures 1A and 1B). Concurrent blood pressure recording indicated animal stability during the procedure (Figures 1A and 1B, green recordings). Changes in the CVNA were observed as higher burst count and quantified as number of integrated signal peaks in a time bin of analysis (Figures 1A and 1B, right panels, and Figure 1C for quantitative analysis).

### Bioelectronic Stimulation of the Celiac Vagus Nerve Activates Splenic Nerve Firing

**Figure 2:**
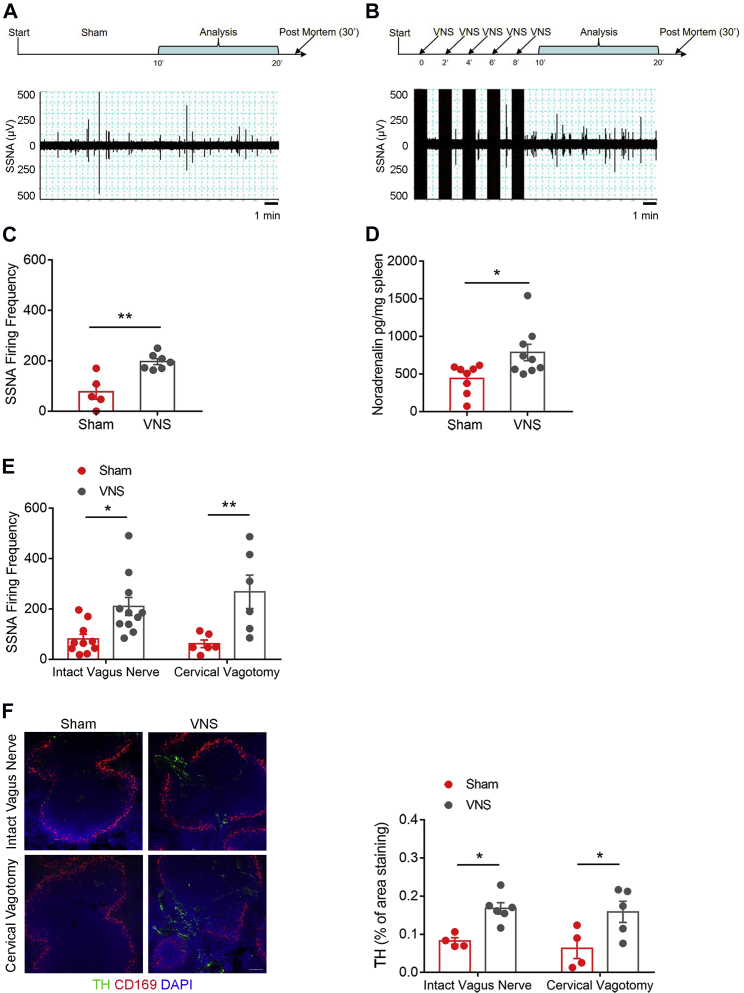
Celiac Vagus Nerve Stimulation Evokes Splenic Nerve Activity and Recruits the Splenic Adrenergic Pathway (A–C) Representative splenic nerve recordings during either sham (A) or VNS (B) procedure. As highlighted in the timelines above the recordings, sham and VNS procedures had a common time window of analysis between 10 and 20 min. VNS stimulation is performed every other minute between the starting point and 10 min. Sham-operated non-stimulated mice were subjected to the same surgical protocol, but the stimulator was kept off (see Figure S2A, related to Figure 2, for the experiment’s schematics). Mice subjected to VNS increased splenic nerve firing frequency (C) (t(10) = 4.298, ^∗∗^p < 0.01). Data were obtained from n = 5 sham and n = 7 VNS mice and are represented as mean ± SEM. (D) Levels of noradrenaline were assessed using ELISA in spleen homogenates and were significantly increased in mice subjected to VNS (t(15) = 2.575, ^∗^p < 0.05) Data were obtained from n = 8 sham and n = 9 VNS mice and are represented as mean ± SEM. (E) In order to abolish potential confounding effects of an afferent reflex vagal response, in a further experimental group, the same VNS protocol was applied in mice that were previously subjected to cervical vagus nerve resection or to the sham procedure (see Figure S2B, related to Figure 2, for the experiment’s schematics). The activity of the splenic nerve after VNS was increased both with intact cervical branch of the vagus nerve and with denervated cervical nerve (intact nerve: sham versus VNS, q(29) = 4.035, ^∗^p < 0.05; cervical vagotomy: sham versus VNS, q(29) = 4.877, ^∗∗^p < 0.01). Data were obtained from n = 10 sham intact vagus, n = 11 VNS intact vagus, n = 6 sham cervical vagotomy, and n = 6 VNS cervical vagotomy mice and are represented as mean ± SEM. (F) TH-positive staining significantly increased in the spleen of mice subjected to VNS, with both intact and resected cervical vagus nerve (intact nerve: sham versus VNS, q(15) = 4.122, ^∗^p < 0.05; cervical vagotomy: sham versus VNS, q(15) = 4.433, ^∗^p < 0.05). Data were obtained from n = 4 sham intact vagus, n = 6 VNS intact vagus, n = 4 sham cervical vagotomy, and n = 5 VNS cervical vagotomy mice and are represented as mean ± SEM (scale bar, 100 μm).

Our next investigation sought to understand how bioelectronically stimulating the celiac vagus nerve affected splenic nerve activity and the downstream immune response. In order to accomplish this, we applied bioelectronic stimulation to the celiac efferent of the vagus nerve and simultaneously recorded the resulting splenic nerve firing (Figure S2A, related to Figure 2). We positioned two different pairs of electrodes, one in recording mode placed on the splenic nerve and the second in stimulating mode placed on the celiac vagus nerve (Figure S3, related to Figure 2). For 10 min, we applied vagus nerve stimulation (VNS) every other minute, and then we recorded 10 min of SSNA in the subsequent time bin, as shown in the schematics in Figures 2A and 2B. The control mice were prepared with the same procedure, but VNS was not applied (Figures 2A and 2B). Ten minutes of VNS resulted in significantly increased SSNA in the subsequent 10 min time bin, compared with sham non-stimulated mice (Figures 2A and 2B and Figure 2C for quantitative analysis). *Ex vivo* analysis of the spleen revealed effective upregulation of the adrenergic pathway in mice subjected to VNS. Quantitatively measured noradrenaline levels, which are detected using ELISA in spleen homogenates, were notably higher in mice subjected to VNS compared to sham-operated, non-stimulated mice (Figure 2D).

One key function of the vagus nerve is controlling reflex responses through central actions mediated by its cervical trunk (Mancia and Grassi, 2014). In order to rule out the afferent vagus nerve’s potential involvement in the effects observed when stimulating the celiac efferent branch, we analyzed another animal group, in which VNS- or sham-treated mice underwent either a cervical vagotomy or a control surgical procedure before starting the stimulation-recording protocol (Figure S2B, related to Figure 2). VNS significantly raised SSNA in mice with severed cervical vagus nerve, in a way overlapping the effect observed in mice with intact cervical vagus nerve (Figure 2E). In addition, the immunofluorescence analysis showed a marked increase of TH innervation, labeled in green, in the marginal zone delineated by CD169-positive macrophages, stained in red (Figure 2F). This change was not linked to the presence of an intact cervical vagus nerve (Figure 2F), similar to what was observed for functional effect.

### Vagal Stimulation Increases PlGF Expression and Immune Activation inside the Spleen

**Figure 3:**
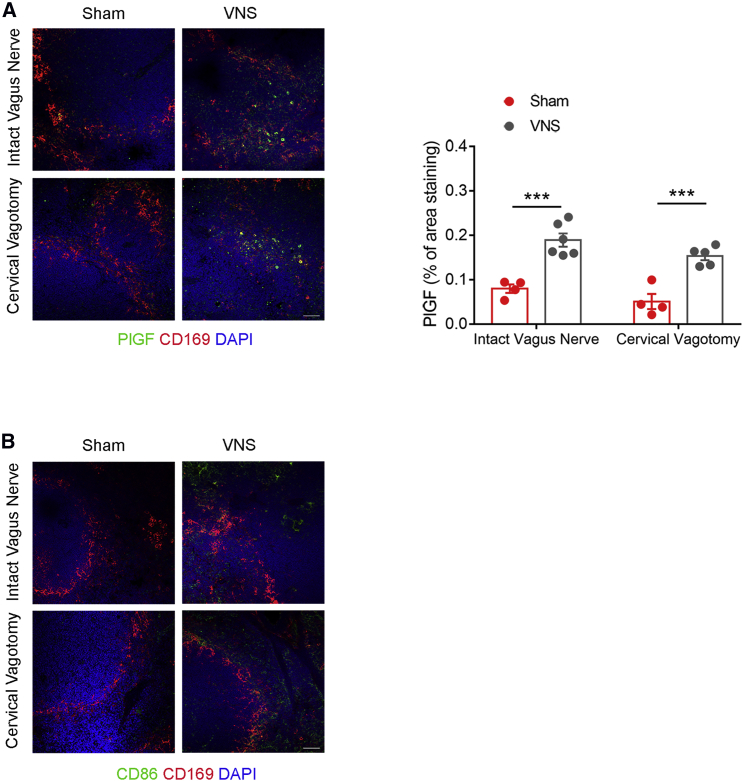
VNS Increases PlGF Expression and CD86 Co-stimulation Factor in the Spleen (A) VNS significant upregulated splenic PlGF expression, measured as the area stained in green, and localized in the marginal zone delimited by CD169-positive macrophages red. The increase of PlGF expression was observed both with intact and resected cervical vagus nerve (intact nerve: sham versus VNS, q(15) = 8.112, ^∗∗∗^p < 0.001; cervical vagotomy: sham versus VNS, q(15) = 7.322, ^∗∗∗^p < 0.001). Data were obtained from n = 4 sham intact vagus, n = 6 VNS intact vagus, n = 4 sham cervical vagotomy, and n = 5 VNS cervical vagotomy mice and are presented as mean ± SEM (scale bar, 50 μm). (B) VNS induced the expression of the co-stimulation molecule CD86, stained in green. CD169 marker was used to delineate the marginal zone macrophages in red (scale bar, 50 μm).

At the molecular level, we found that bioelectronic stimulation of the vagus nerve celiac efferent increased PlGF expression in the spleen’s marginal zone, as evidenced by immunofluorescent labeling for PlGF in green and marginal zone CD169 macrophages in red, with either intact or resected cervical vagus nerve (Figure 3A, representative images on the left and quantitative analysis on the right). At the same time, VNS promoted the activation CD86 expression (Figure 3B), a typical hallmark of T cell co-stimulation and hence a key indicator of fully activated adaptive immune response, which is necessary for eventual activated cell recruitment toward peripheral tissues. Similar to what was observed for PlGF expression, the effect of CD86 upregulation was independent of the presence of an intact cervical vagus nerve (Figure 3B).

**Figure 4:**
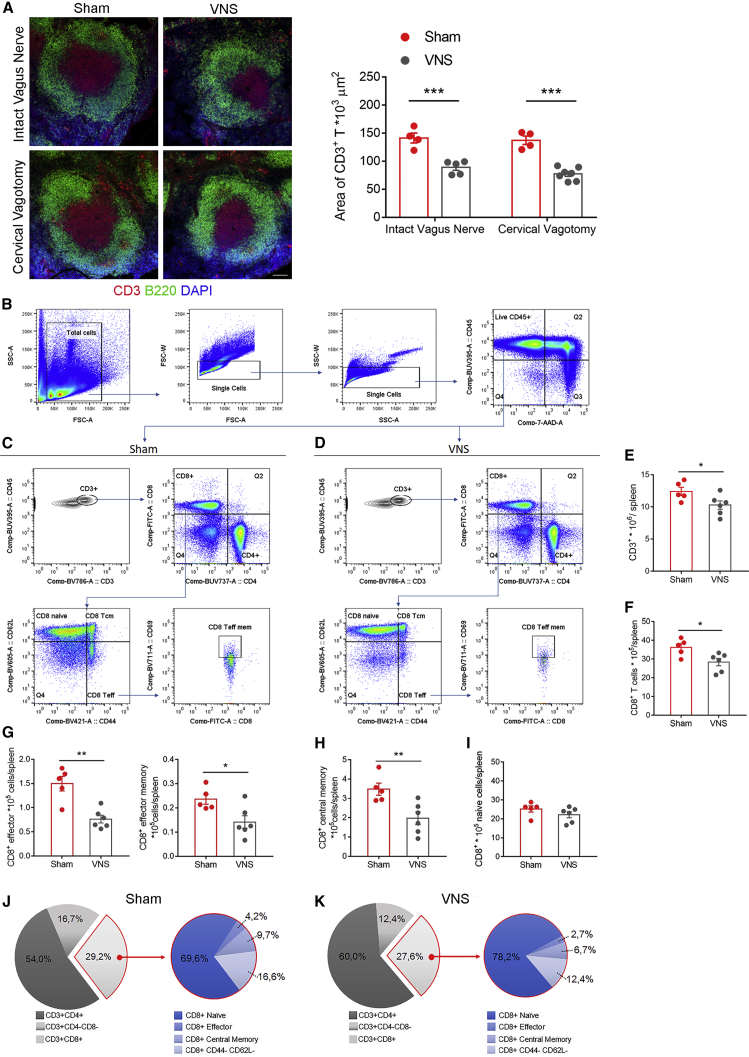
VNS Activates T Cell Egress from the Spleen and Shows a Selective Ability to Promote Deployment of CD3^+^CD8^+^ Effector Subsets of Cells (A) VNS induced T cell egress from the spleen, as evidenced by a reduced area of CD3^+^ cells (red), delimited by B220^+^ cells (green) depicting the B cell area, independently from the integrity of cervical vagus nerve (intact nerve: sham versus VNS, q(15) = 7.867, ^∗∗∗^p < 0.001; cervical vagotomy: sham versus VNS, q(15) = 9.392, ^∗∗∗^p < 0.001). Data were obtained from n = 4 sham intact vagus, n = 5 VNS intact vagus, n = 4 sham cervical vagotomy, and n = 7 VNS cervical vagotomy mice and are presented as mean ± SEM (scale bar, 100 μm). (B–K) Flow cytometry analysis was used to investigate the specific T cell subpopulations recruited by VNS, according to the gating strategy shown in the representative plots in (B)–(D). Among the overall reduction of CD3^+^ cells (t(9) = 2.325, ^∗^p < 0.5) (E), only CD3^+^CD8^+^ cells (F) were significantly diminished after VNS (t(9) = 2.759, ^∗^p < 0.05), while CD3^+^CD4^+^ count remained unaltered (graph not shown; t(9) = 1.044, p > 0.05). By gating out on the CD3^+^CD8^+^ subpopulations, we evidenced a marked reduction in CD3^+^CD8^+^CD44^+^CD62L^−^ effector cells (G) (t(9) = 4.509, ^∗∗^p < 0.01), CD3^+^CD8^+^CD44^+^CD62L^−^CD69^+^ effector resident memory cells (G) (t(9) = 2.752, ^∗^p < 0.05), and CD3^+^CD8^+^CD44^+^CD62L^+^ central memory cells (H) (t(9) = 3.3, ^∗∗^p < 0.01), while CD3^+^CD8^+^CD44^−^CD62L^+^ naive cells (I) showed no difference (t(9) = 1.285, p > 0.05). Data were obtained from n = 5 sham and n = 6 VNS mice and are presented as mean ± SEM. VNS-stimulated mice showed a redistribution of the CD8 subpopulation frequencies (J) and (K), resulting in the absolute reduction of effector and memory cells.

VNS also promoted a reduction in T cell content in the splenic reservoir, as shown by smaller CD3-positive T cell area in the white pulp, compared with sham-operated, non-stimulated mice (Figure 4A, representative panels on the left and graph with quantitative analysis on the right). Both co-stimulation and reduction of T cell content in the spleen, induced by VNS, occurred with or without an intact cervical vagus nerve (Figures 3B and 4A, respectively), thereby suggesting that the celiac vagus nerve directly promotes immune activation in the spleen.

Next, we conducted flow cytometry on spleen single-cell preparations, using the gating strategy in Figures 4B–4D, to assess the effect of VNS on the specific lymphocyte subsets. Interestingly, we found that despite an overall decrease in total CD45^+^CD3^+^ cells (Figure 4E), a selective reduction occurred for CD45^+^CD3^+^CD8^+^ (Figure 4F) but not CD45^+^CD3^+^CD4^+^ lymphocytes (graph not shown; statistics reported in the figure legend). In addition, by gating on the CD45^+^CD3^+^CD8^+^ subpopulations, we found notably fewer effector (Teff: CD44^+^CD62L^−^; Figure 4G), effector memory (Teff mem: CD44^+^CD62L^−^CD69^+^; Figure 4G), and central memory (Tcm: CD44^+^CD62L^+^; Figure 4H) cells, while naive (CD44^−^CD62L^+^; Figure 4I) cells showed only a non-significant trend of reduction. Last, the analysis of variation in proportions of CD8 T cells subpopulations similarly highlighted a remarkable reduction of the effector fraction of CD8 T cells (Figures 4J and 4K).

In order to further investigate whether the VNS-induced T cell reduction was due to egress of cells toward peripheral tissues or apoptosis/death inside the spleen, we evaluated variations in the circulating blood pool of CD8 T cells and apoptosis/cell death in splenic cells. Multiparametric flow cytometry was performed on circulating leukocytes according to the gating strategy shown in Figure S4A (related to Figure 4). We found a significant increase in the subpopulation of effector CD8^+^ (Teff: CD44^+^CD62L^−^; Figure S4A) in mice subjected to VNS. At the same time, apoptosis and cell death were simultaneously evaluated in the spleen, demonstrating that the level of apoptotic (Hoecst^hi^-7AAD^int^; Figure S4B, related to Figure 4) and dead (Hoecst^hi^-7AAD^hi^; Figure S4B) cells, identified by the gating strategy shown in Figure S4B, were comparable in mice subjected to VNS or to sham procedure. Taken together these results indicated that VNS promoted egress of CD8 T effector cells in the circulation, toward peripheral tissues.

### PlGF Is a Key Mediator of the Neuroimmune Interface in the Spleen

**Figure 5:**
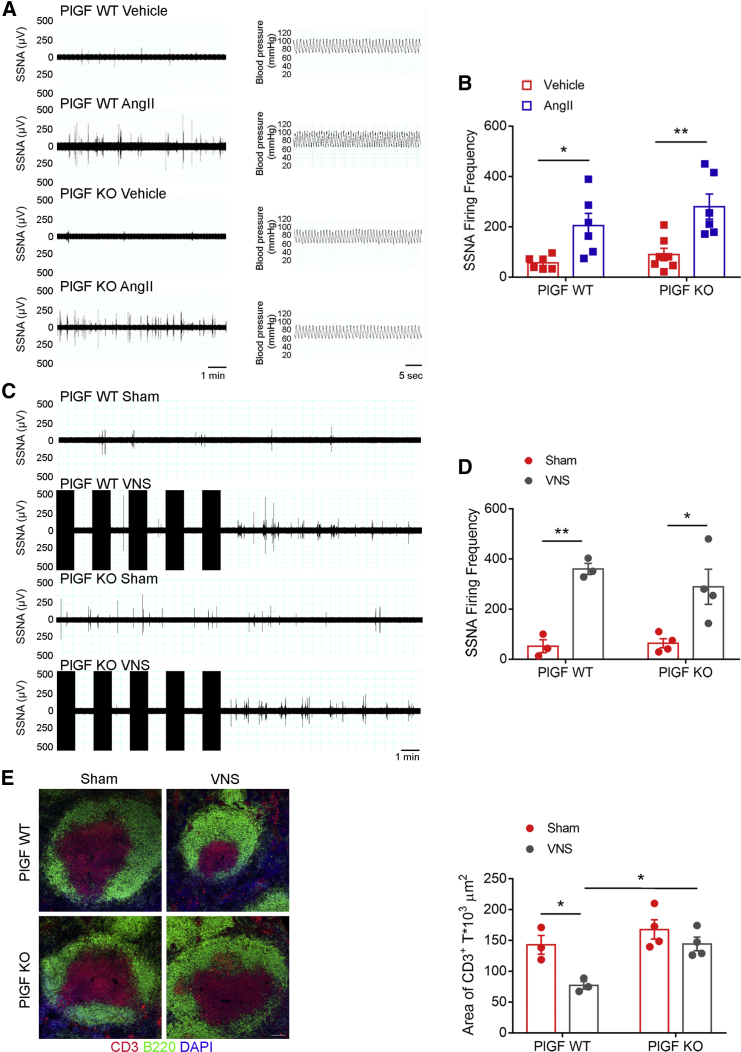
PlGF Plays a Dispensable Role in the VNS-Induced Neural Transmission from the Vagus Nerve to the Spleen but Has a Crucial Role in Promoting the Ensuing Immune Activation in the Spleen (A) Representative raw signals of SSNA in a time bin of 10 min in WT and PlGF-deficient mice subjected either to AngII infusion or vehicle. (B) PlGF-deficient mice show the same SSNA response to AngII infusion observed in WT mice, as evidenced in the quantitative analysis of firing frequency (WT, vehicle versus AngII: q(21)] = 3.983, ^∗^p < 0.05; PlGF-deficient, vehicle versus AngII: q(21) = 5.307, ^∗∗^p < 0.01). Data were obtained from n = 6 vehicle WT, n = 6 AngII WT, n = 7 Veh PlGF-deficient, and n = 6 AngII PlGF-deficient mice and are presented as mean ± SEM. (C and D) WT and PlGF-deficient mice were subjected to either sham or VNS procedure, resulting in a comparable increase in SSNA (WT, sham versus VNS: q(10) = 6.392, ^∗∗^p < 0.01; PlGF-deficient, sham versus VNS: q(10) = 5.392, ^∗^p < 0.05). Data were obtained from n = 3 sham WT, n = 3 VNS WT, n = 4 sham PlGF-deficient, and n = 4 VNS PlGF-deficient mice and are presented as mean ± SEM. (E) In PlGF-deficient mice, the VNS is uncapable of promoting T cell egress, as evidenced by the area of CD3^+^ cells (red) that shows no variation compared with sham non-stimulated mice (WT sham versus VNS, q(10) = 4.616, ^∗^p < 0.05; WT VNS versus PlGF-deficient VNS, q(10) = 5.038, ^∗^p < 0.05; PlGF-deficient sham versus VSN, q(10) = 1.916, p > 0.05). Data were obtained from n = 3 sham WT, n = 3 VNS WT, n = 4 sham PlGF-deficient, and n = 4 VNS PlGF-deficient mice and are presented as mean ± SEM (scale bar, 100 μm).

To determine PlGF’s mechanistic role in activating the splenic adaptive immune response evoked by VNS, we gave PlGF-deficient mice chronic AngII infusion for 3 days and then assessed SSNA. We found that nerve activity firing was comparable with that measured in control wild-type (WT) mice (Figure 5A, left panels, and Figure 5B, quantitative measurements). As previously observed, the blood pressure recordings were stable during the procedure, thus excluding an effect on SNS related to blood pressure variations (Figure 5A, right panels).

Similarly, bioelectronic stimulation of the celiac vagus nerve (i.e., the VNS procedure) produced a comparable SSNA increase in PlGF-deficient and WT mice (Figures 5C and 5D for representative stimulation/recording patterns and quantitative analysis, respectively), suggesting that the absence of PlGF does not affect neural signal transduction from the celiac vagus nerve to the splenic nerve. However, despite inducing nerve activation, VNS in PlGF-deficient mice failed to promote T cell egress from the spleen. As shown by the area of CD3^+^ lymphocytes estimated in the various experimental groups, PlGF-deficient mice subjected to VNS did not have reduced T cell area, instead showing measurements comparable with those from sham-operated, non-stimulated mice and significantly greater area than that observed in WT mice subjected to VNS (Figure 5E, representative images on left and quantitative analysis on right). Taken together, the above data suggest PlGF is not necessary to neural signal transmission from the vagus nerve to the spleen but does play a fundamental role in transducing the neuronal impulse that reaches the spleen to generate an immune response.

### VNS-Induced PlGF Release and Immune Response Initiation Are Mediated by α-Adrenergic Receptor Signaling

**Figure 6:**
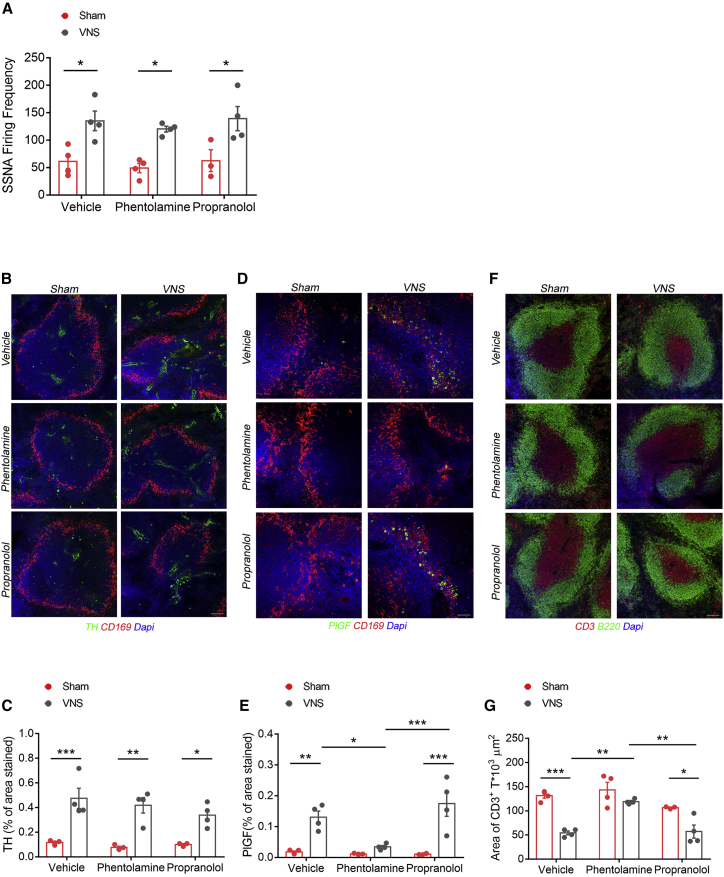
VNS-Induced PlGF Release and Immune Response Initiation Are Mediated by α-Adrenergic Receptor Signaling (A–C) Vehicle-, phentolamine-, or propranolol-treated mice were subjected to either sham or VNS procedure. (A) SSNA (vehicle, sham versus VNS: q(17) = 4.936, ^∗^p < 0.05; phentolamine, sham versus VNS: q(17) = 4.753, ^∗^p < 0.05; propranolol, sham versus VNS: q(17) = 4.729, ^∗^p < 0.05) and (B and C) TH-positive staining (vehicle, sham versus VNS: q(17) = 7.480, ^∗∗∗^p < 0.001; phentolamine, sham versus VNS: q(17) = 7.118, ^∗∗^p < 0.01; propranolol, sham versus VNS: q(17) = 4.599, ^∗^p < 0.05) (scale bar, 100 μm) were increased in all VNS groups. (D and E) Although vehicle- and propranolol-treated mice shown a similar activation of PlGF, phentolamine treatment significantly reduced it (vehicle, sham versus VNS: q(17) = 5.856, ^∗∗^p < 0.01; propranolol, sham versus VNS: q(17) = 7.861, ^∗∗∗^p < 0.001; VNS, phentolamine versus vehicle: q(17) = 4.991, ^∗^p < 0.05; VNS, phentolamine versus propranolol: q(17) = 7.361, ^∗∗∗^p < 0.001) (scale bar, 50 μm). (F and G) A reduced area of CD3^+^ cells (red), delimited by B220^+^ cells (green), is observed in both vehicle- and propranolol-treated VNS mice. Conversely, phentolamine-treated VNS mice display a CD3^+^ area comparable with sham mice (vehicle, sham versus VNS: q(17) = 8.337, ^∗∗∗^p < 0.001; propranolol, sham versus VNS: q(17) = 4.955, ^∗^p < 0.05; VNS, phentolamine versus vehicle: q(17) = 6.983, ^∗∗^p < 0.01; VNS, phentolamine versus propranolol: q(17) = 6.688, ^∗∗^p < 0.01) (scale bar, 100μm). For all panels, data were obtained from n = 4 sham vehicle, n = 4 VNS vehicle, n = 4 sham phentolamine, n = 4 VNS phentolamine, n = 3 sham propranolol, and n = 4 VNS propranolol mice and are represented as mean ± SEM.

Overall, the above observations suggest that PlGF release depends on noradrenergic signaling in the spleen. To further elucidate the underlying mechanisms we first interrogated the ImmGen database (Immunological Genome Project, 2020) to verify whether both α- and β-adrenergic receptors are expressed by splenic cells. As both α- and β-adrenoreceptors are widely expressed in the spleen, we planned an experimental setting aimed at blocking one or another receptor before subjecting mice to VNS or sham procedure. In order to avoid confounding effects of a generalized α- or β-adrenergic inhibition, we devised an experimental strategy to directly deliver the α- or β-blockers in the spleen, through a micro-infusion of phentolamine or propranolol, respectively, in the splenic artery, 30 min before applying the VNS stimulation or the sham procedure. We did not observe any differences in splenic nerve firing frequency, ruling out the influence of α- or β-adrenoreceptor blockade on the nerve signal transmission between the celiac vagus nerve and the splenic nerve (Figure 6A). Similarly, TH immunofluorescence showed that VNS, in the presence of phentolamine or propranolol, induced effects comparable with those observed with vehicle alone (Figure 6B). When we analyzed the effects of VNS on PlGF expression (Figure 6C) and CD3 T cell area (Figure 6D), phentolamine pre-treatment hampered PlGF upregulation and T cell egress, while propranolol pre-treatment had no effect on both parameters, thus suggesting that PlGF activation and consequent splenic immune response is dependent on an α-adrenergic signaling recruited by VNS.

## Discussion

We investigated the effects of chronic exposure to the peptide hormone AngII on vagus nerve activation and splenic immune response. Our findings show that AngII activates increased burst firing of the vagus nerve celiac efferent and that this activation is strictly related to noradrenergic stimulation in the spleen and adaptive immunity priming. In addition, in the absence of AngII infusion, we reproduced its effects by bioelectronically stimulating the celiac vagus nerve. Applying an alternate electronic stimulation protocol to the celiac end of the vagus nerve triggered sympathetic activity in the splenic nerve. The noradrenergic stimulation permeated the spleen and, by releasing noradrenaline in the marginal zone, induced the α-adrenoreceptor mediated activation of the growth factor PlGF, which was previously found out at the splenic neuroimmune interface in hypertension.

AngII is known to promote the activation of T cell co-stimulation mediated by CD86 (Vinh et al., 2010). Accordingly, we have previously shown that splenic production of PlGF, induced by AngII (Carnevale et al., 2014), is associated with lymphocyte maturation before they egress from lymphoid organs, such as the spleen, and move toward peripheral tissues. Our present study demonstrated that bioelectronic stimulation of the celiac vagus nerve promotes PlGF and CD86 expression in the splenic marginal zone by evoking nerve activity and activating the noradrenergic pathway. More important, the observed effect was coupled with T cell egress from the spleen; interestingly, however, egress was not generalized among all T cells but rather limited to select subpopulations. In fact, bioelectronic stimulation of the celiac vagus nerve promoted the egress of CD8 T cells but not CD4 T cells. Among the CD8 T cells, the effector subsets diminished in the spleen after VNS, while concomitantly increasing in the pool of circulating leukocytes. Thus, the vagal/splenic component, challenged either directly by bioelectronics or indirectly by AngII, modulates the immune repertoire mainly by controlling the deployment of effector CD8 T cells, which are known to exert a major role in the cardiovascular injury that follows chronic hypertension. It should be noted that although our data demonstrate that VNS stimulates CD8 T effector cells egress, in a way similar to that observed upon hypertensive challenges, the capability of bioelectronic stimulation to modulate the process of antigen-dependent CD8 T subset differentiation remains to be deciphered. Future studies will be necessary to provide evidence that modulating splenic nerve activity also regulate antigen-specific adaptive immune responses.

This work also provides evidence that although the presence of PlGF is dispensable for transmitting the AngII- or bioelectronic stimulation-recruited signal from the vagus to the splenic nerve and hence into the spleen, it is necessary for shifting the noradrenergic pathway toward an effective splenic immune response and, at the same time, depends on a noradrenergic signaling in the spleen. Mice genetically ablated for PlGF showed no defect in evoking the splenic noradrenergic pathway when either infused with AngII or stimulated directly on the celiac vagus nerve. However, despite successful recruitment of the splenic noradrenergic pathway, PlGF-deficient mice failed to prime adaptive immunity and deploy T cells toward peripheral organs. Conversely, the dependence of PlGF activation on the noradrenergic input arriving through the vagus-splenic nerve was demonstrated. We selectively blocked the α- and β-adrenergic receptors to elucidate which class of receptors could be the one triggering PlGF release, finding that mice subjected to selective splenic α-adrenergic blockade were unable to release PlGF in response to VNS and to initiate the process of the T cell deployment. On the contrary, when the β-adrenergic blocker propranolol was used, no effect was observed neither on PlGF expression or on T cell egress. Taken together, these data definitively identify PlGF as a critical molecular neuroimmune transducer in the spleen, coupling sympathetic nerve activity with adaptive immune response assembly through an α-adrenergic dependent mechanism in the spleen. Future studies will elucidate which cell type in the spleen produces PlGF upon noradrenergic input and which cell type expresses the cognate receptors transducing the PlGF-mediated neuroimmune effects. The finding that PlGF expression is quite uniquely restricted to the marginal zone, where TH fibers entangle the structures involved in T cell circulation and egress (Chauveau et al., 2020), suggests that its signaling could be involved in mediating T cells migration processes inside the spleen.

The splenic nerve firing and noradrenergic pathway activation obtained by stimulating the celiac vagus nerve resembled those generated by chronic exposure to AngII. It has been clearly demonstrated that AngII pro-hypertensive actions are mediated by the central nervous system. In fact, the disruption of specific brain areas sensitive to circulating AngII hampered blood pressure increase and activation of the correlated adaptive immune response (Marvar et al., 2010). Also, our previous work showed that AngII enhances sympathetic drive in the splenic district only when the celiac vagus nerve remains intact, allowing immune activation and blood pressure increase (Carnevale et al., 2016). Hence, on the basis of the results of the present work, we can reasonably assert that the reflex response recruited by AngII in the brain is conveyed from the brain to the spleen through the coupling of the celiac vagus and splenic nerves at the level of the celiac ganglion.

In conclusion, the finding that bioelectronic stimulation of the celiac vagus nerve promotes the egress of a selective subpopulation of effector CD8 T cells, in the absence of any other challenge, offers an opportunity to start thinking of bioelectronic medicine as an innovative tool to fine-tune immune responses in various pathophysiological contexts. Although our findings fit well with previous observations obtained in the context of hypertension, specific bioelectronic stimulation protocols may be able to modulate splenic immunity. One future challenge will be to find appropriate bioelectronic stimulation protocols capable of generating the desired immune functions in clinical conditions wherein effector CD8 T cells may help counteract disease progression. CD8 T cells are known to exert their effects mainly through two mechanisms: cytolytic activities against target cells or cytokines and secretion of various chemokines (Lugli et al., 2020). The possibility of influencing the immune response toward a CD8 T effector profile via bioelectronic approaches, rather than immunomodulating drugs with many other undesirable effects, could be beneficial for promoting antitumor activity or enhancing resistance against infection by various pathogens.

## STAR★Methods

### Key Resources Table

**Table:** 

REAGENT or RESOURCE	SOURCE	IDENTIFIER
**Antibodies**		

BUV395 Rat Anti-Mouse CD45 Clone 30-F11	BD Biosciences	Cat# 564279; RRID:AB_2651134
BV786 Hamster Anti-Mouse CD3e Clone 145-2C11	BD Biosciences	Cat# 564379; RRID:AB_2738780
FITC Rat Anti-Mouse CD8a Clone 53-6.7	BD Biosciences	Cat# 553031; RRID:AB_394569
BUV737 Rat Anti-Mouse CD4 Clone RM4-5	BD Biosciences	Cat# 564933; RRID:AB_2732918
PE-CF594 Rat Anti-Mouse CD25 Clone PC61	BD Biosciences	Cat# 562694; RRID:AB_2744346
BV421 Rat Anti-Mouse CD44 Clone IM7	BD Biosciences	Cat# 563970; RRID:AB_2738517
BV605 Rat Anti-Mouse CD62L Clone MEL-14	BD Biosciences	Cat# 563252; RRID:AB_2738098
BV711 Hamster Anti-Mouse CD69 Clone H1.2F3	BD Biosciences	Cat# 740664; RRID:AB_2740352
Purified Rat Anti-Mouse CD45R/B220 Clone RA3-6B2	BD Biosciences	Cat# 550286; RRID:AB_393581
Purified Hamster Anti-Mouse CD3 Clone 145-2C11	Biorad	Cat# MCA2690T; RRID:AB_1101793
Purified Rat Anti-Mouse CD169 Clone 3D6.112	Biorad	Cat# MCA884; RRID:AB_322416
Purified Rabbit Anti-Mouse PlGF	Abcam	Cat# Ab9542; RRID:AB_307330
Purified Rabbit Anti-Mouse CD86 Clone EP1158Y	Novus Biologicals	Ca-t# NB110-55488; RRID:AB_837989
Purified Sheep Anti-Mouse Tyrosine Hydroxylase	Millipore	Cat# AB1542; RRID:AB_90755
AlexaFluor 488 Donkey Anti-Rat IgG (H+L)	Jackson Immunoresearch	Cat# 712-545-153; RRID:AB_2340684
Cy3-AffinityPure Goat Anti-Hamster IgG (H+L)	Jackson Immunoresearch	Cat# 127-165-099; RRID:AB_2338988
Cy3-AffinityPure Goat Anti-Rat IgG (H+L)	Jackson Immunoresearch	Cat# 112-165-003; RRID:AB_2338240
Biotinylated anti-Rabbit in Horse	Vector Laboratories	Cat# BA-1100; RRID:AB_2336201
Streptavidin AlexaFluor488 coniugate	Invitrogen	Cat# S32354; RRID:AB_2315383
AlexaFluor488 AffinityPure Donkey Anti-Rabbit IgG (H+L)	Jackson Immunoresearch	Cat# 711-545-152; RRID:AB_2313584
Cy3-AffinityPure Donkey Anti-Sheep IgG (H+L)	Jackson Immunoresearch	Cat# 713-165-003; RRID:AB_2340727

**Critical Commercial Assays**		

Noradrenaline ELISA Kit	IBL international	Cat# RE59261

**Chemicals, Peptides, and Recombinant Proteins**		

Angiotesin II human, ≥ 93%	Sigma Aldrich	Cat# A9525
7-AAD	BD Biosciences	Cat# 559925
Hoecst 33423	Invitrogen	Cat# H3570
DAPI-containing medium	Thermo Fisher Scientific	Cat# 62248
Donkey Serum	Sigma Aldrich	Cat# D9663
Normal Goat Serum Blocking Solution	Vector Laboratories	Cat# S-1000
Normal Horse Serum Blocking Solution	Vector Laboratories	Cat# S-2000
Polyvinyl alcohol mounting medium with DABCO	Sigma Aldrich	Cat# 10981
Propranolol	Sigma Aldrich	Cat# P0884
Phentolamine	Sigma Aldrich	Cat# P7547

**Experimental Models: Organisms/Strains**		

Mouse: *Pgf*^*−/−*^ (indicated as PlGF-deficient)	Gigante et al., 2006	N/A
Mouse: C57BL/6J	Charles River	RRID:IMSR_JAX:000664

**Software and Algorithms**		

ImageJ	NIH	RRID:SCR_003070
GraphPad Software	PRISM7	RRID:SCR_002798
SPSS 23.0 Software	IBM	RRID:SCR_002865
BD FACSDiva Software	BD Biosciences	RRID:SCR_001456
FlowJo V10.0.8 Software	Tree Star Inc.	RRID:SCR_008520
MATLAB	Mathworks	RRID:SCR_001622
LabChart 7	ADInstruments	N/A
ImmGen	ImmGen Consortium	N/A

### Resource Availability

#### Lead contact

Further information and request for resources should be directed to the Lead Contact, Giuseppe Lembo (lembo@neuromed.it).

#### Materials availability

This study did not generate new unique reagents or mouse lines.

#### Data and code availability

Dataset available upon request to the Lead Contact, Script for CVNA processing is publicly available: https://github.com/LorCarnevale/CVNA_Process

### Experimental Model and Subject Details

Experimental procedures were carried out according to the EC Council Directive 2010/63 and Italian legislation on animal experimentation (Decreto Legislativo D.Lgs 26/2014). All procedures were designed and performed to minimize animal suffering while respecting the principles of the “three Rs” (Replacement, Reduction, Refinement). All experiments were performed on male C57BL/6J mice, obtained from Charles River, and inbred Placental Growth Factor (PlGF) deficient mouse and wild-type littermates, all aged 8-12 weeks (Carnevale et al., 2011; Gigante et al., 2006). Mice were housed under controlled temperature (21 ± 1°C) and relative humidity (60 ± 10%), with a 12-12 hour dark-light cycle, sawdust as bedding, pellet food and water *ad libitum*.

### Method Details

#### Angiotensin II infusion

Mice, anaesthetized with 5% isoflurane and maintained with 1%–1.5% with 1L per minute of oxygen on a homeothermic blanket to keep body temperature between 37 ± 0.5°C, underwent surgery to implant subcutaneous osmotic minipumps infusing AngII (0.5 mg kg−1day−1; Sigma Aldrich) or vehicle (NaCl 0.9%).

#### Nerve recording and stimulation – surgery

Stainless steel electrodes were obtained from MLA1214 Spring Clip Electrodes (ADInstruments) and refined to fit the space under the splenic or celiac vagus nerve. Electrodes were connected to Animal Bio Amp (ADInsturments), a digital amplifier and a sampler with an internal gain of factor 10,000, set for sampling at 4kHz (doubling the minimum frequency of sampling needed to avoid aliasing). Stimulating electrodes were obtained from ADInstruments and wired to the Stimulus Isolator (ADInstruments), a current-controlled stimulator. Both amplifier and stimulator were serially connected to an 8/35 Power Lab Acquisition system (ADInstruments). Blood pressure monitoring was performed using a single tip-pressure catheter (Millar, SPR-100) placed in the left femoral artery and connected to a pressure transducer interface (Millar, MPVS ULTRA). Two input channels were set on microneurographic signal and blood pressure signal, one output channel was set on the nerve stimulator. The PowerLab was interfaced with a personal computer running LabChart 7 (ADInstruments) to collect, record and monitor all data.

Procedures for splenic sympathetic nerve activity (SSNA) recordings were performed as previously described (Carnevale et al., 2016), and a similar protocol for recording or stimulating the celiac vagus nerve was established as follows. Mice were anaesthetized with 5% isoflurane and maintained with 1%–1.5% with 1L per minute of oxygen. A homeothermic blanket maintained body temperature between 37 ± 0.5°C. The splenic district was exposed via an abdominal incision followed by moving aside the intestine. Once the spleen and splenic artery were visible, the splenic nerve was isolated, and the recording electrodes were placed beneath it. After stabilizing the nerve signal, silicone gel was placed on the electrodes for complete isolation. The same incision and splanchnic district exposure procedure was performed to record or stimulate the celiac vagus nerve. Then, we moved aside the surrounding tissue further to expose the celiac ganglion and celiac branch distal end of the vagus nerve. To record celiac vagus nerve activity (CVNA), the recording electrodes were placed under the nerve and isolated after reading nervous activity. In the experimental setup for vagus nerve stimulation (VNS), after placing the recording electrodes on the splenic nerve, the stimulating electrodes were placed under the celiac branch of the vagus nerve. After verifying that the signal on the splenic nerve remained unchanged before stimulation, silicone gel was added for further isolation, as described above.

Procedures for vehicle, phentolamine or propranolol injection in the splenic artery were performed after exposing the splenic artery before the positioning of the electrodes. The splenic artery was cannulated as previously described (Carnevale et al., 2014) and connected to an infusion syringe pump. 100μl of vehicle or treatment solution were injected in the splenic artery in 10 minutes.

All the described procedures were performed in a faraday cage containing the surgical desk and all the microneurographic equipment, allowing an absolute isolation from the surrounding electric noise.

#### Nerve recording and stimulation – analysis

Data collection was performed using LabChart 7 (ADInstruments). SSNA analysis was performed using the Spike Analysis Module of LabChart, as previously described (Carnevale et al., 2016). The splenic nervous signal was pre-processed to highlight the frequencies of interest (300-1,000 Hz band pass filtering, with a further 50Hz notch filter to eliminate the noise generated by electric cord current). The resulting signal was expressed in μV and spikes identified in this way were categorized as spikes of intensity greater than the baseline noise, estimated from post-mortem recordings. The firing frequency was defined as the number of spikes in a 10-minute time bin of analysis. The amplitude gain was obtained as the ratio between spike peak and baseline noise in a time bin.

CVNA was analyzed with LabChart and MATLAB (Mathworks). The raw signal was recorded with the same setting used for the splenic nerve but filtered with a stricter band pass (300-550 Hz) to avoid high-frequency noise. The filtered signal was integrated with a 0.1 s time-constant decay and exported into MATLAB for peak analysis. Hence, each activation ensemble, comprising several spikes, was combined and independently counted as a single activation burst. CVNA was quantified as number of bursts in a 10-minute time bin of analysis.

VNS was performed according to the following protocol: the vagus nerve was stimulated every other minute within a 10-minute stimulation window, with a stimulation frequency of 5 Hz, and a stimulation pattern generated by a monophasic pulse of 0.3 mA.

#### Vagotomy

Left celiac vagotomy was performed while recording SSNA. The celiac branch of the vagus nerve was exposed as described for recording electrode positioning. A silk suture thread was knotted to the distal end of the nerve. The nerve was excised by pulling the thread between the first and the second time bins. To ensure optimal signal stability throughout the entire experiment, no further manipulations of the splenic district occurred.

In another group of mice, left cervical vagotomy was performed before recording the SSNA and stimulating the celiac branch of the vagus nerve. During the preparation for electrophysiological recordings, a midline cervical incision exposed the left vagus trunk, which was carefully separated from the carotid artery and then cut with surgical forceps while starting the recordings on the splenic nerve. The cervical vagotomy resects the nerve in a very apical region of the nerve, alongside the carotid. Leaving intact the celiac-mesenteric branch where the stimulation is performed.

#### Tissue Isolation

At the end of each recording experiment, the spleen was excised and isolated for further analysis. For immunofluorescence analyses, the spleens were embedded in OCT and stored at −20°C for subsequent cryo-sectioning. For biochemical analyses, the spleens were flash-frozen in liquid nitrogen and stored at −80°C. For flow cytometry, the spleens were isolated and immediately processed for single-cell suspension preparation and analysis, the blood was collected from the carotid artery in EDTA-tubes and immediately processed for single-cell suspension preparation and analysis.

#### Immunofluorescence analysis

Spleen sections were obtained with a cryostat microtome (Leica 1950CM, Leica Microsystems). 25 μm sections were cut and post-fixed in 4% PFA for 15 minutes and subsequently processed for staining.

The following primary antibodies were used: tyrosine hydroxylase (TH), CD169, CD3, CD45R/B220, CD86, PlGF. Resulting sections were incubated with secondary antibodies conjugated to Alexa Fluor 488 and Cy3 or with biotinylated antibody incubated with streptavidin Alexa Fluor 488 conjugate. DAPI was used to counterstain nuclei and, after processing, slides were coverslipped (DABCO, Sigma Aldrich).

Slides were scanned using a Zeiss 780 confocal laser-scanning microscope, with a 405nm Diode laser to excite DAPI, a 488nm argon laser to excite Alexa Fluor 488 and a 543nm HeNe laser to excite Cy3. The resulting images, with pseudocolors attributed according to the description in figure legends, were processed for quantitative analysis as previously described (Carnevale et al., 2018; Da Ros et al., 2017), analyzing 3-9 field of views from at least 3 different sections for each animal. Intermodes thresholding was used to quantify the area stained for TH and PlGF stainings and data are reported as average value for single mouse.

For analyzing the area of T cells in the spleen we measured the area of CD3+ staining surface as a measure of total lymphocyte content in the spleen with ImageJ (NIH) in 5-9 field of view from 5 different sections per mice, reporting the average value for single mouse.

#### Noradrenaline assay

Spleen were minced in an extraction buffer containing 0.1 HCl and 1mM EDTA and assayed with a high-sensitivity ELISA kit (IBL International, Hamburg, Germany), according to the manufacturer’s instructions. Results are presented as pg mg^-1^ of wet tissue.

#### Flow Cytometry

After the VNS/Sham procedure, the spleens were excised and directly analyzed for flow cytometric analysis of lymphocytes. Single-cell suspensions were obtained as previously described (Carnevale et al., 2014, 2016; Perrotta et al., 2018).

1 × 10^6^ splenic leukocytes were preincubated with anti-CD16/32 Fc receptor (BD Bioscience) and then incubated with the following primary antibodies (all from BD Biosciences): BUV395-anti-CD45 (BD564279), BV786-anti-CD3 (BD564379), FITC-anti-CD8 (BD553031), BUV737-anti-CD4 (BD564933), PE-CF594-anti-CD25 (BD562694), BV421-anti-CD44 (BD563970), BV605-anti-CD62L (BD563252), BV711-anti-CD69 (BD740664). An additional incubation with the live/dead marker 7-AAD (BD559925) was performed. Data from the entire sample were acquired on a FACS Celesta flow cytometer with FACS Diva software (BD Biosciences) and analyzed using FlowJo software (V10.0.8, Tree Star).

Apoptosis analysis was performed by flow cytometry utilizing the double staining of immune cells with 7-AAD (BD559925) and Hoechst 33342 (Invitrogen H3570). 1 × 10^6^ splenic leukocytes were preincubated with anti-CD16/32 Fc receptor (BD Bioscience) and then incubated with the following primary antibodies (all from BD Biosciences): BV786-anti-CD3 (BD564379), FITC-anti-CD8 (BD553031), PE-anti-CD4 (BD553049). An additional incubation of 7 minutes at 37°C with Hoechst 33342 and then a 10 minutes incubation at 4°C with 7-AAD. Both markers were used at the concentration of 1μg/mL.

Blood samples were collected into microvette with EDTA. Leukocytes extracted from the blood were preincubated with anti-CD16/32 Fc receptor (BD Bioscience) and then incubated with the following primary antibodies (all from BD Biosciences): BUV395-anti-CD45 (BD564279), BV786-anti-CD3 (BD564379), FITC-anti-CD8 (BD553031), BUV737-anti-CD4 (BD564933), PE-CF594-anti-CD25 (BD562694), BV421-anti-CD44 (BD563970), BV605-anti-CD62L (BD563252), BV711-anti-CD69 (BD740664). An additional incubation with the live/dead marker 7-AAD (BD559925) was performed. Data from the entire sample were acquired on a FACS Celesta flow cytometer with FACS Diva software (BD Biosciences) and analyzed using FlowJo software (V10.0.8, Tree Star).

### Quantification and Statistical Analysis

Sample size estimation was performed by power analysis, based on experiments previously carried out in our laboratory and from published literature. All data are presented as mean ± standard error. Shapiro-Wilk test was used to assess data distribution normality, while Levene’s test was used to assess equality of variances. Statistical significances were assessed with Student t test on two groups of data with normal distributions, two-way ANOVA with Tukey’s post hoc was used in multiple group analysis. All the above-mentioned analyses were carried out with SPSS 23.0 (IBM Software) and graphical representations produced with PRISM7 (GraphPad Software).

## Supplemental Information

Document S1. Figures S1–S4

Document S2. Article plus Supplemental Information
