# Supplementary material for: Celiac Vagus Nerve Stimulation Recapitulates Angiotensin II-Induced Splenic Noradrenergic Activation, Driving Egress of CD8 Effector Cells
Source: Cell Rep. 2020 Dec 15;33(11):108494. doi: 10.1016/j.celrep.2020.108494 (PMC7758159; doi:10.1016/j.celrep.2020.108494)
Supplement: Document S1. Figures S1–S4 [file mmc1.pdf]

Cell Reports, Volume *33*

Supplemental Information

Celiac Vagus Nerve Stimulation Recapitulates Angiotensin II-Induced Splenic Noradrenergic Activation, Driving Egress of CD8 Effector Cells

Lorenzo Carnevale, Fabio Pallante, Marialuisa Perrotta, Daniele Iodice, Sara Perrotta, Stefania Fardella, Francesco Mastroiacovo, Daniela Carnevale, and Giuseppe Lembo

A

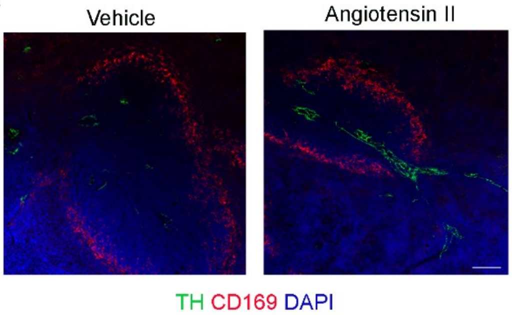

B

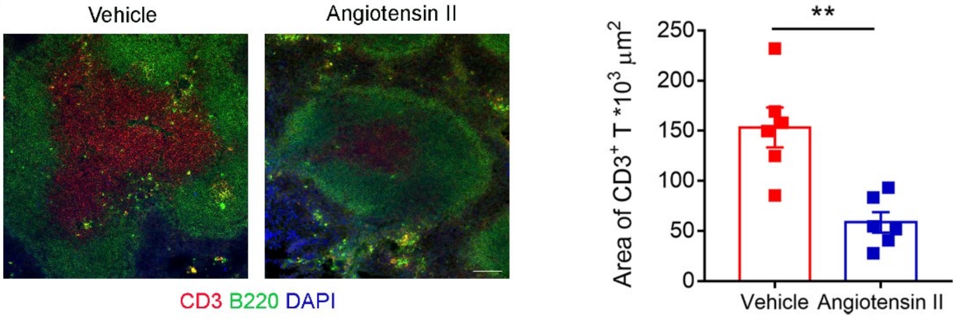

Figure S1 related to Figure 1

Figure S1 related to Figure 1. AngII increases the expression of TH in the spleen and promotes T cell egression in a pre-hypertensive phase. **(A)** AngII significantly enhances the TH positive innervation in the spleen and, at the same time (Scalebar 100µm), **(B)** induces T cells egression from the spleen, assessed as CD3+ area (red), delimited by B cell area marked as B220+ cells (green) ($t(10)=4.229$, $^{**}P<0.01$) (Scalebar 100µm). All data are represented as mean ± SEM.

A

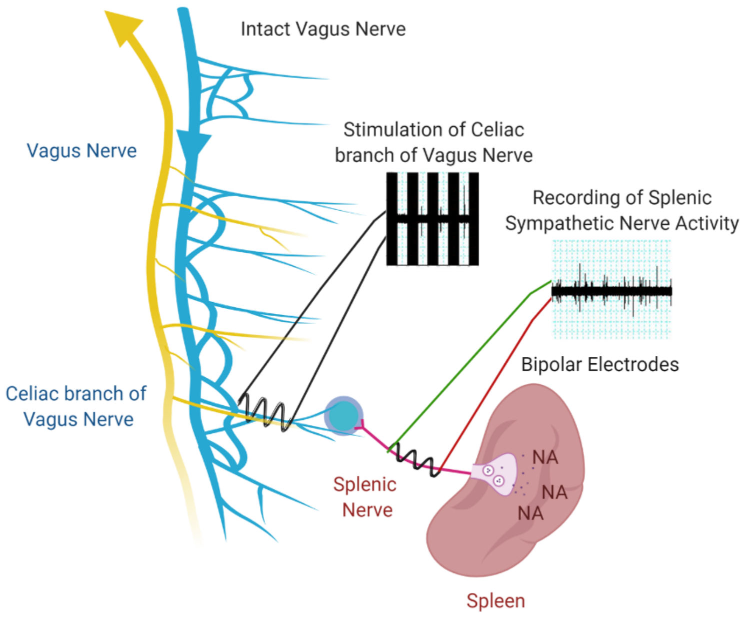

B

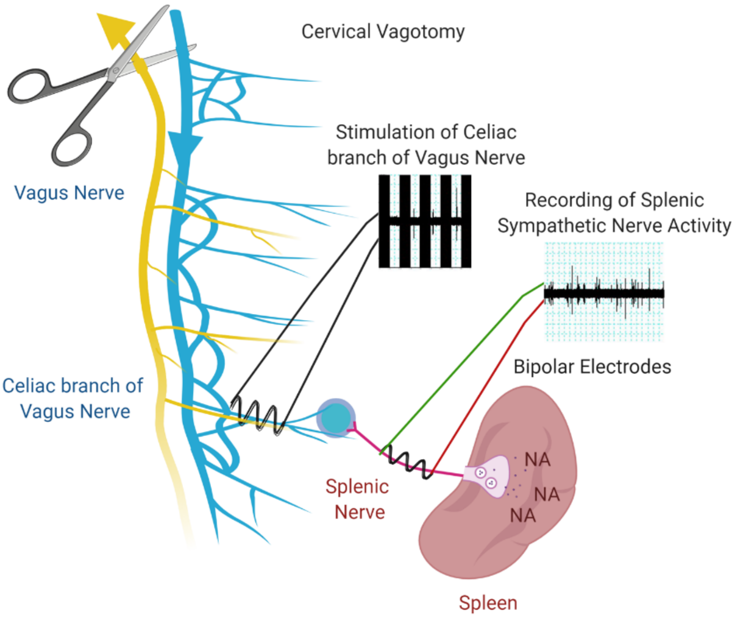

Figure S2 related to Figure 2

Figure S2 related to Figure 2. Schematics of the experimental setup for concurrent nerve stimulation and recording. **(A)** Schematics of the experimental setup for celiac vagus nerve stimulation (VNS) with concurrent splenic sympathetic nerve activity (SSNA) recording. (**B**) Schematics of the same experimental setup, performed in mice subjected to surgical resection of cervical branch of the vagus nerve or sham procedure.

A

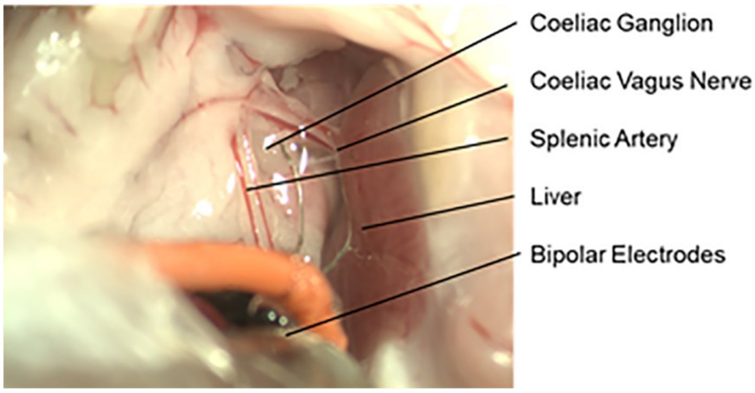

Figure S3 related to Figure 2

Figure S3 related to Figure 2. (A) Representative image of the surgical procedures. The bipolar stimulating electrodes are placed under the coeliac vagus nerve, highlighted and exposed in the current picture. The second couple of recording electrodes are placed under the splenic artery, to record the signal from the splenic nerve.

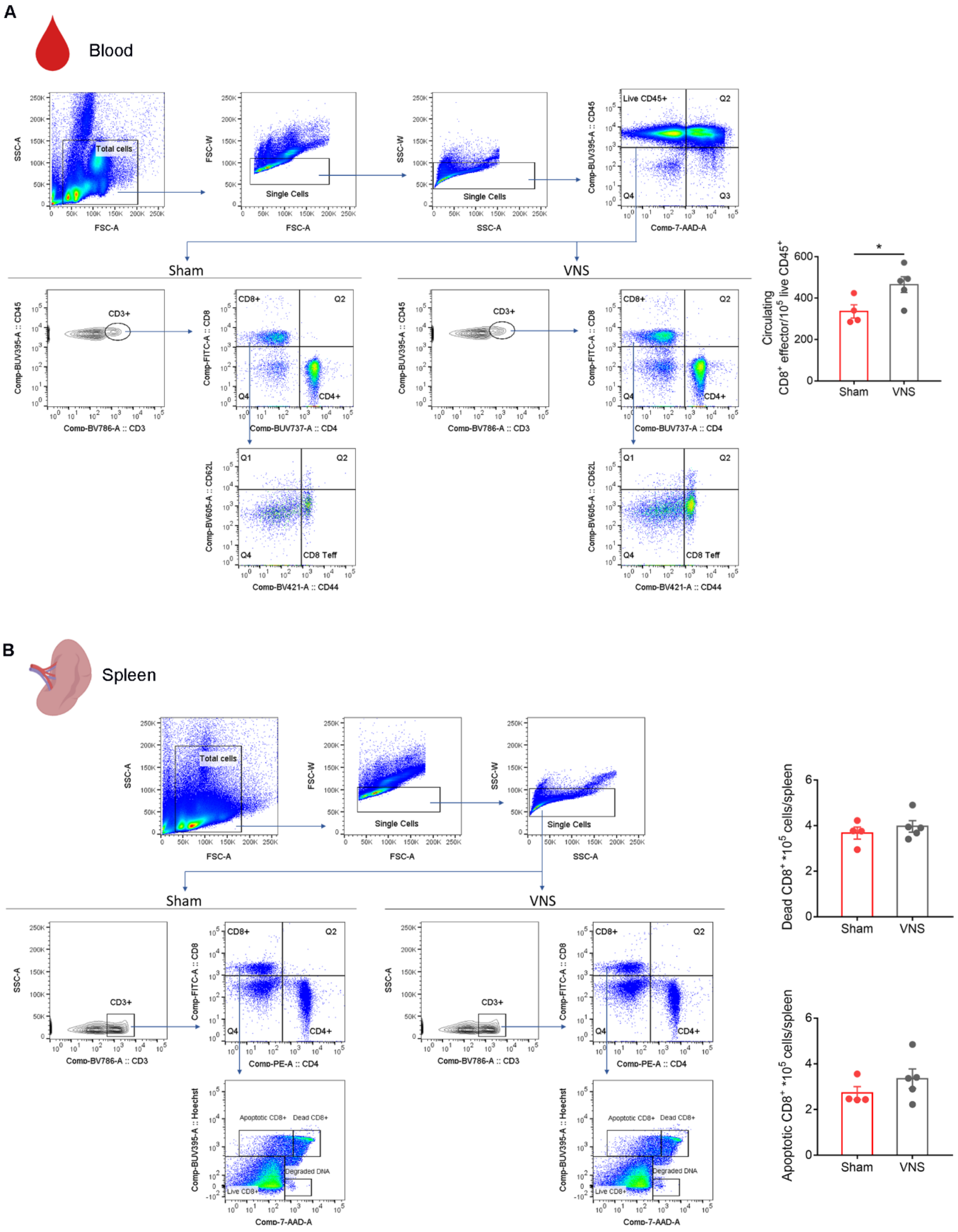

Figure S4 related to Figure 4

Figure S4 related to Figure 4. (A) Representative gating scheme for flow cytometry analysis of blood. The quantitative analysis of $CD8^+$ effector cells shows an increased number of circulating effector cells in stimulated mice ($t(7)=2.522$, $*P<0.05$). **(B)** Representative gating scheme for flow cytometry analysis of splenic apoptosis. The quantitative analysis of dead and apoptotic $CD8^+$ cells shows no differences between sham and stimulated mice (dead: $t(7)=0.790$, $P=0.455$; apoptotic: $t(7)=1.145, P=0.289$). All data are represented as mean ± SEM.
